# Comment on ‘BAG-1 as a biomarker in early breast cancer prognosis: a systematic review with meta-analyses'

**DOI:** 10.1038/s41416-018-0023-z

**Published:** 2018-03-15

**Authors:** Willi Sauerbrei, Tim Haeussler

**Affiliations:** Institute for Medical Biometry and Statistics, Faculty of Medicine and Medical Center, University of Freiburg, Freiburg, Germany

**Keywords:** Biomarkers, Breast cancer

Prognostic biomarkers play an important role in medical research, assessment of prognosis and treatment of patients. Validation of a potential biomarker takes a long evidence-based approach requiring several individual studies, a systematic review of the literature and a meta-analysis.^1^ Papadakis et al.^2^ conducted a systematic review with meta-analysis to assess BAG-1 as an effective prognostic biomarker in early breast cancer. They concluded ‘Meta-analyses suggested improved outcome with high BAG-1 […] expression’. They identified 18 papers providing results from 20 studies (Table 2 in Papadakis et al.^2^), assessed the quality of reporting according to the REMARK guidelines,^3, 4^ summarised key results of each study (Table 2 in Papadakis et al.^2^) and conducted three meta-analyses (Figure 2 in Papadakis et al.^2^). This study illustrates key steps required for an evidence-based biomarker assessment; however, we have identified several major weaknesses in the assessment of the quality of reporting and the meta-analyses. We concluded that results and inferences from this study are not justified by the assessments and analyses presented.

## Assessment of the quality of reporting according to REMARK

According to Table 1 in Papadakis et al.^2^, reporting of the studies was excellent, strongly contradicting the results of a recent review.^5^ As an example, we consider the rationale for sample size (Item 9) that was positively assessed in all evaluated studies, whereas it was assessed as being adequately reported in only 22, 11 and 8% in the three (sub-)studies (each including about 50 articles), as summarised in Sekula et al.^5^. Statements like ‘All patients […] diagnosed […] between 1995 and 2001, were included [only 70 patients included]’^6^ or ‘292 patients diagnosed […] between February 1992 and August 2002’^7^ (see also Papadakis et al^2^) are not sufficient as rationale. The authors do not cite the REMARK Explanation and Elaboration paper^3^ and may not have been aware that more details are required.

As all studies consider a survival-time outcome (time to event analysis), it is necessary to provide the number of events as a measure of the effective sample size.^3^ Under the term ‘Study power’ (Item 9), the sample size is listed but not the number of events.

Item 16 of the REMARK guidelines states that ‘For key multivariable analyses, report estimated effects (for example, hazard ratio) with confidence intervals for the marker and, at least for the final model, all other variables in the model.’ Sekula et al.^5^ found acceptable reporting in 70, 66 and 62% of studies assessed. Except for one study, all BAG-1 studies were positively assessed. However, in the manuscript by Papadakis et al^2^, several studies reported only *p* values^8, 9^ or otherwise indicated non-significance^10^. Significant shortcomings in reporting the primary studies are also found in overviews and hamper any sensible assessments of a marker’s effect. Instead of the positive assessments of reporting quality of the primary studies, Papadakis et al.^2^ should have clearly pointed out the weaknesses. How can a study be positively assessed if it mentions a significant effect in univariate analysis, shows a Kaplan–Meier plot illustrating large survival differences (Figures 4a, 4c in Athanassiadou et al. ^6^) without providing an estimate of the effect? Furthermore, in multivariable analysis, the effect seems to be non-significant.

## Meta-analysis

Papadakis et al.^2^ state ‘In general, data were too heterogeneous, and outcome measures were too varied to perform meta-analyses for the majority of studies. Meta-analyses of mRNA expression from the two data sets analysed in Millar et al.^7^ and the data set analysed in Papadakis et al.^11^ including a total of 2422 patients produced an HR of 0.55 (95% CI: 0.36–0.85) favouring improved BCSS with high expression of BAG-1 (Figure 2a in Papadakis et al.^2^).’ The first sentence implies that a sensible meta-analysis is not possible, an unfortunate situation seen in many systematic reviews of prognostic biomarkers. Nevertheless, Papadakis et al.^2^ conducted three meta-analyses summarising two or three studies. It is seriously misleading to pick out a small number of studies and pretend to conduct a meta-analysis. In addition, they averaged effects for classification of BAG-1 as ‘positive’ vs ‘negative’, but criteria for this classification varied substantially (positivity was defined as ‘H-Score >100,’^10^ and ‘dichotomised at a cutoff value of 40% positively staining nuclei at any intensity’^7^ (see also Papadakis et al^2^)). They also combined hazard ratios from univariate and multivariable analyses (Figure 2c in Papadakis et al.^2^).

In three meta-analyses, the authors consider two methods of measuring BAG-1 and two outcomes. Of the 18 papers identified, 14 are ignored in these ‘meta-analyses’. Can that be described as a meta-analysis?

## Meaningful meta-analyses of biomarkers: individual participant data required

In two meta-analyses (Figure 2a, b in Papadakis et al.^2^), estimates from univariate models from the original studies are averaged. Because associations of a biomarker with outcome might be heavily influenced by other prognostic variables correlated with the biomarker, such univariate analyses can be seriously misleading^3^ (Item 15). An appropriate analysis of a single study requires a multivariable model in which the effect of interest is adjusted for potential confounders. Consequently, a suitable meta-analysis requires averaging of adjusted effects that can be combined meaningfully. Collaboration among several study groups and individual participant data are required to make populations ‘broadly’ comparable and to reanalyse individual studies so as to give adjusted effects that can be validly combined.^1, 12^ We are well aware of the serious difficulties encountered in conducting such a project. However, many researchers and collaborative groups have realised their necessity and IPD meta-analyses of prognostic factors have become more popular during the past decade.^13^

## Publication bias and the need for a comprehensive biomarker study registry

Publication bias distorts the estimate of the true value of biomarkers. This important issue needs to be considered in studies trying to assess the value.^1, 12, 14^ Unfortunately, Papadakis et al.^2^ do not even mention this issue. As for randomised trials, several researchers argue for establishing a comprehensive biomarker study registry^12, 15, 16^.

In conclusion, in light of previous experience, we are not surprised to see so many weaknesses in reports of individual studies and in meta-analyses. The complexity of such projects is often underrated and the importance of including an experienced methodologist is still not recognised sufficiently. In addition, evidence-based assessments require substantial collaboration among researchers, including willingness to share data. The paper and the situation criticised for BAG-1 in breast cancer is not much different from the situation today for many other prognostic biomarkers. Fortunately, many researchers understand the difficulties, and several promising projects have been started, e.g., the PROGRESS partnership, which provides a framework to improve prognostic factor research (progress-partnership.org). We hope that by writing this letter, we can lessen the chance that others will make basic errors in future studies and point researchers to key steps needed when assessing the prognostic value of a biomarker. As biomarkers become more and more important tools in medical decision-making, invalid results can have serious consequences for patients.
